# TLR8 reprograms human Treg metabolism and function

**DOI:** 10.18632/aging.102223

**Published:** 2019-09-04

**Authors:** Xia Liu, Lingyun Li, Guangyong Peng

**Affiliations:** 1 Division of Infectious Diseases, Allergy and Immunology, Department of Internal Medicine, Saint Louis University School of Medicine, Saint Louis, MO 63104, USA

**Keywords:** Treg cells, metabolism, glycolysis, TLR8, cancer immunotherapy

T regulatory cells (Tregs) play a critical role in maintaining immune tolerance and homeostasis, but they are an obstacle for effective immunity against cancer and chronic infections. Increasing evidence suggests that different T cell subsets require distinct energetic and biosynthetic pathways to perform their specific and functional immune responses. Studies from mouse models have shown that murine Tregs have low levels of the glucose transporter Glut1 but with a high rate of lipid oxidation, while Th1, Th2, and Th17 cells are highly glycolytic with high levels of Glut1 [1]. However, human Tregs have distinct metabolic profiles with murine Tregs. Freshly-isolated human Tregs are highly glycolytic and they require both fatty acid oxidation and glycolysis for their proliferation and functions [2,3]. We have more recently demonstrated distinct metabolic profiles of human Tregs compared with effector T cells [3]. Our studies clearly suggest that both human naturally occurring Tregs (nTregs) and tumor-derived Tregs exhibit more active glucose metabolism than the other types of T cells (Th1, Th2 and Th17 cells), showing high expression levels of Glut1, Glut3, and glycolytic enzymes, as well as more active glucose uptake [3]. In addition, human Tregs mainly depend on glucose metabolism to execute their suppressive functions [3].

It is well-established that Treg cells induce an immunosuppressive microenvironment that is a major obstacle for successful tumor immunity and immunotherapy. Progress has been made in understanding the molecules and mechanisms that Treg cells use to mediate immune suppression in different models, including inhibitory cytokines and secreted molecules, cytolysis or apoptosis of target cells, consumption of limiting growth factors and metabolic disruption. However, the precise suppressive mechanisms utilized by human Tregs are unclear and need to be further clarified [4,5]. We identified that human nTregs and tumor-derived γδ Tregs induce responder T cell senescence as a novel suppressive mechanism [5,6]. We further identified that Treg-mediated heightened glucose consumption induced an increased phosphorylation of AMPK and DNA damage response in responder T cells during their cross-talk, resulting in cell senescence and dysfunction in responder effector T cells [4]. Therefore, development of novel strategies to metabolically control Treg functionality in the tumor microenvironment is critical for effective anti-tumor immunity and immunotherapy.

**Figure 1:**
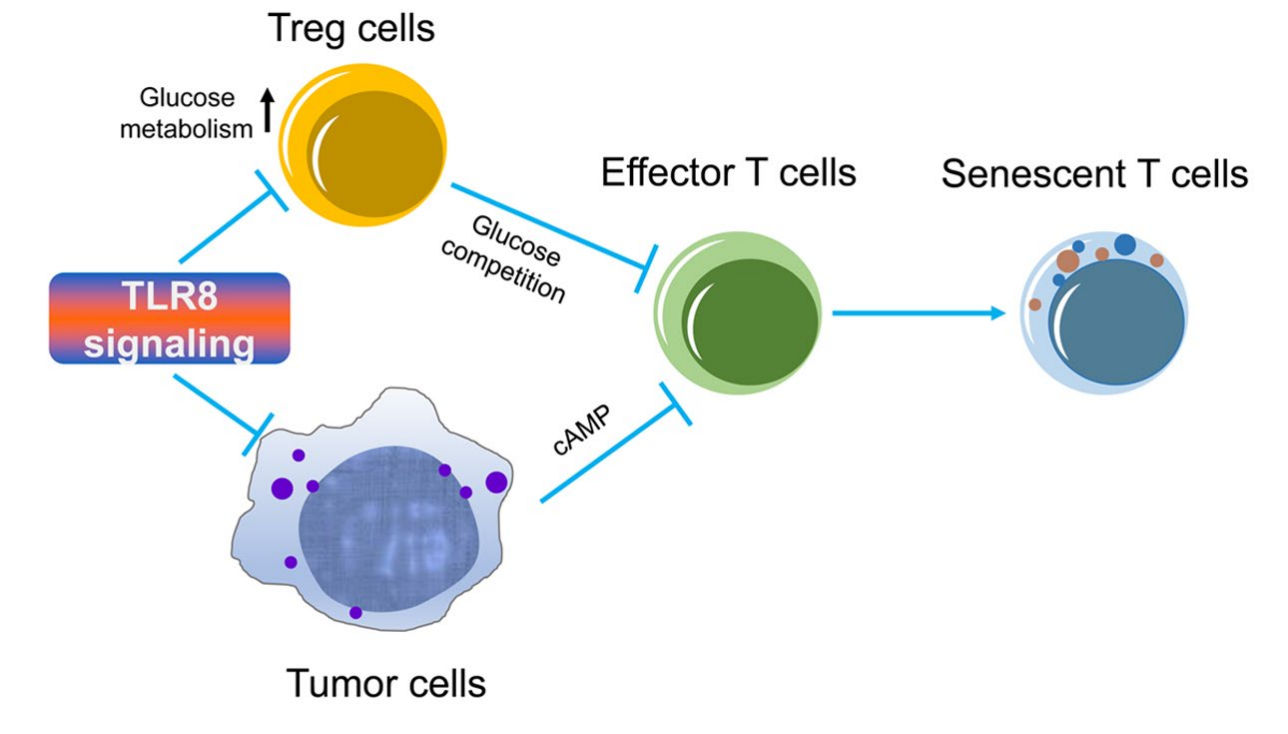
**TLR8 signaling enhances anti-tumor immunity and immunotherapy.** Treg cells and tumor cells have accelerated glucose metabolism and induce senescence and suppression in effector T cells during their cross-talks. TLR8 signaling inhibits glucose metabolism in Treg cells and decreases tumor-produced cAMP, resulting in reversal of responder T cell senescence and enhanced anti-tumor immune responses.

Toll-like receptors (TLRs) are important for innate immunity and inflammatory responses, acting as a bridge between innate and adaptive immunity. Increasing evidence suggests that TLR signaling influences the development, differentiation and function of T-cell subsets. TLR signaling also directly regulates energy metabolism in different types of immune cells. It has been shown that activation of TLR1 and TLR2 signaling in mouse Tregs can increase Treg glycolysis and proliferation but reduces their suppressive capacity [7]. Our recent studies showed that human TLR8 signaling, but not other TLRs, directly reversed the suppressive function and senescence induction mediated by human nTregs, as well as tumor-derived CD4^+^, CD8^+^ T cells, and γδ Treg cells [3–5]. Mechanistically, we discovered that TLR8 signaling can selectively inhibit human Treg cell metabolism, resulting in reversal of their suppressive activity [3] (Figure 1). TLR8 activation in Tregs directly suppresses glucose uptake and glycolysis of Treg cells via downregulation of Gluts and key glycolytic enzymes. Furthermore, TLR8 signaling molecularly down-regulates mTOR-HIF1α axis and targets glycolytic programs in human Treg cells, which reprograms Treg glucose metabolism and inhibitory functions. In addition, in vivo studies using T cell adoptive transfer therapy models indicate that TLR8 signaling can specifically inhibit Treg glucose metabolism, resulting in prevention of effector T cell senescence and enhancing antitumor immunity [3]. These studies provide the novel concept that reprogramming of glucose metabolism and function in human Treg cells via TLR8 signaling is a potentially effective strategy for tumor immunotherapy.

In addition to immune cells, TLRs are also widely expressed on tumor cells, regulating tumor growth and function. Our previous studies have demonstrated that different types of human tumor cells directly induce T cell senescence and dysfunction due to the tumor-derived endogenous metabolite cyclic adenosine monophosphate (cAMP) [8]. However, activation of TLR8 signaling in tumor cells can decrease cAMP levels in tumor cells and dramatically promote responder tumor-specific T cell antitumor efficacy (Figure 1). Our studies collectively suggest that TLR8 signaling can reprogram both Treg and tumor cell metabolism and functions, resulting in the reversal of effector T cell senescence and dysfunctional state within the tumor microenvironment and enhanced anti-tumor immune responses for tumor immunotherapy. Given that limited success rates of current immunotherapies have been shown in the clinical cancer patients, including checkpoint blockade immunotherapy with anti-PD1/PDL1 and anti-CTLA4, TLR8-mediated reprogramming of the metabolism and functions in Treg and tumor cells is an important and alternative immunotherapeutic strategy for cancer therapy. Future studies should focus on the strategies using TLR8-mediated metabolic reprogramming of the tumor microenvironment, combined with adoptive T-cell therapy and/or checkpoint blockade therapy that target both tumor and T cells, to improve antitumor immune responses for immunotherapy in different tumor models.
